# Inhibitor of Cysteine Protease of *Plasmodium malariae* Regulates Malapains, Endogenous Cysteine Proteases of the Parasite

**DOI:** 10.3390/pathogens11050605

**Published:** 2022-05-22

**Authors:** Hương Giang Lê, Jung-Mi Kang, Tuấn Cường Võ, Thảo Dương Nguyễn, Myunghwan Jung, Min Kyoung Shin, Won Gi Yoo, Byoung-Kuk Na

**Affiliations:** 1 Department of Parasitology and Tropical Medicine, and Institute of Health Sciences, Gyeongsang National University College of Medicine, Jinju 52727, Korea; gianglee291994@gmail.com (H.G.L.); jmkang@gnu.ac.kr (J.-M.K.); vtcuong241@gmail.com (T.C.V.); baxonguyen@gmail.com (T.D.N.); wgyoo@gnu.ac.kr (W.G.Y.); 2 Department of Convergence Medical Science, Gyeongsang National University, Jinju 52727, Korea; mjung@gnu.ac.kr (M.J.); mkshin@gnu.ac.kr (M.K.S.); 3 Department of Microbiology, and Institute of Health Sciences, Gyeongsang National University College of Medicine, Jinju 52727, Korea

**Keywords:** *Plasmodium malariae*, inhibitor of cysteine protease, malapains, cathepsins, hemoglobin hydrolysis

## Abstract

Cysteine proteases of malaria parasites have been recognized as potential targets in antimalarial drug development as they play pivotal roles in the biology of these parasites. However, strict regulation of their activities is also necessary to minimize or prevent deleterious damage to the parasite and the host. Previously, we have characterized falcipain family cysteine proteases of *Plasmodium malariae*, named as malapains (MPs). MPs are active hemoglobinases. They also may participate in the release of merozoites from mature schizonts by facilitating remodeling of erythrocyte skeleton proteins. In this study, we identified and characterized an endogenous inhibitor of cysteine protease of *P. malariae* (PmICP). PmICP shared similar structural and biochemical properties with ICPs from other *Plasmodium* species. Recombinant PmICP showed a broad range of inhibitory activities against diverse cysteine proteases such as falcipain family enzymes (MP-2, MP-4, VX-3, VX-4, and FP-3), papain, and human cathepsins B and L, with stronger inhibitory activities against falcipain family enzymes. The inhibitory activity of PmICP was not affected by pH. PmICP was thermo-labile, resulting in rapid loss of its inhibitory activity at a high temperature. PmICP effectively inhibited hemoglobin hydrolysis by MPs and regulated maturation of MPs, suggesting its role as a functional regulator of MPs.

## 1. Introduction

Cysteine proteases of malaria parasites have been recognized as potential targets for antimalarial drug development due to their essential roles in the biology of these parasites [1,2]. They are involved in multiple biological processes including cell life cycle and parasite–host cell interactions. Up to now, numerous cysteine proteases have been identified and their biological functions in *Plasmodium* species have been characterized. Particularly, falcipain family enzymes are the most extensively studied ones as attractive targets for new antimalarial drug development [2,3,4]. Falcipain-2 and falcipain-3 are food vacuole cysteine proteases of *Plasmodium falciparum* essentially involved in hemoglobin hydrolysis [5], erythrocyte rupture [6], and merozoite invasion [7]. Meanwhile, falcipain-1 seems to participate in erythrocyte invasion by hepatocyte-derived merozoites or play critical roles in non-erythrocytic parasites [8,9]. Orthologous enzymes of falcipains have also been identified and characterized in other human infecting *Plasmodium* species such as *P. vivax*, *P. malariae* and *P. knowlesi* [10,11,12,13]. They shared highly similar structural, biochemical, and functional properties with falcipains. Therefore, they are classified into falcipain-family enzymes.

Although falcipain family enzymes play pivotal roles in the biology of malaria parasites, strict regulation of their activities is also important to prevent or minimize inadequate superfluous damage to the parasite and host. Endogenous proteins regulating falcipain family enzymes have been investigated in *Plasmodium* species. Falstatin, an endogenous inhibitor of cysteine protease (ICP) of *P. falciparum*, can effectively inhibit falcipains, suggesting that the inhibitor prevents inappropriate activities of parasite and/or host cysteine proteases, thereby facilitating erythrocyte invasion [14]. ICP of *P. berghei* (PbICP) plays important roles in sporozoite invasion to host cells and parasite survival during liver stage development by inhibiting host cell proteases involved in programmed cell death [15]. ICP of *P. yoelii* (PyICP) can regulate proteolytic processes during blood-stage development. It is likely to play a role in liver stage-hepatocyte interactions when exoerythrocytic merozoite is released [16]. These findings suggest that endogenous ICPs of *Plasmodium* parasites play pivotal roles in diverse biological processes of these parasites by regulating both parasite and host cysteine proteases.

Recently, we have characterized malapains (MPs) as falcipain family cysteine proteases of *P. malariae* [13]. MPs are active hemoglobinases of the parasite. They might also be involved in merozoite release from mature schizonts. In this study, we characterized an ICP of *P. malariae* (PmICP) as a counterpart endogenous protein regulating MPs. PmICP is a typical protein of the falstatin family. It effectively inhibited activity and maturation of MPs, suggesting its essential roles in the biology of *P. malariae*.

## 2. Results

### 2.1. Sequence Analysis of the Gene Encoding PmICP

**Figure 1:**
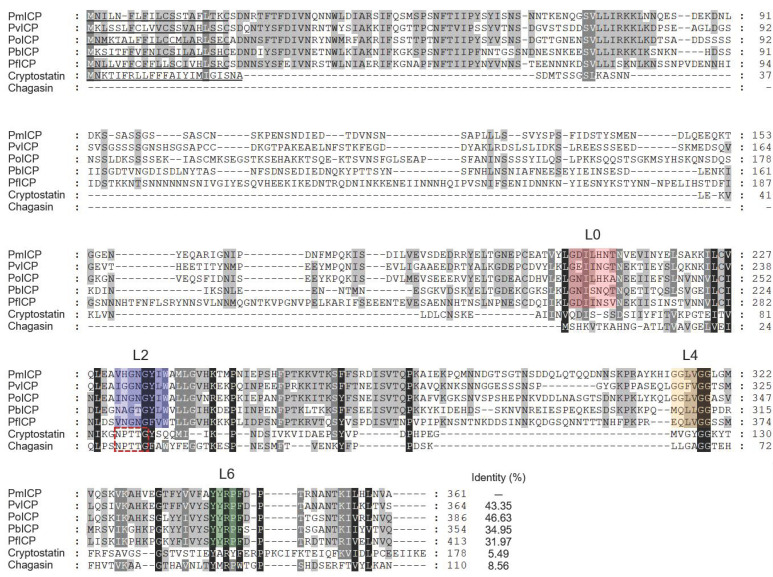
Multiple sequence alignment. Deduced amino acid sequences of ICPs from *Plasmodium* species (PmICP, ICP of *P. malariae*; PvICP, ICP of *P. vivax*; PoICP, ICP of *P. ovale*; PbICP, ICP of *P. berghei*; PfICP, falstatin), cryptostatin (XP_627553.1), and chagasin (Q966X9) were aligned. Dashes represent gaps introduced to maximize alignment. The predicted N-terminal signal sequence is underlined by a black bar. Amino acid residues corresponding to L0, L2, L4, and L6 loops, which are conserved in plasmodial ICPs [17], are shaded with different colors, respectively. The NPTTG motif that is involved in the interaction with catalytic cysteines of target enzymes in the chagasin family proteins is marked by a red dotted box. Percentage of identity among sequences is represented by shading: black (>88%), dark grey (75–88%), light grey (37–75%), and no shading (<37%).

**Figure 2:**
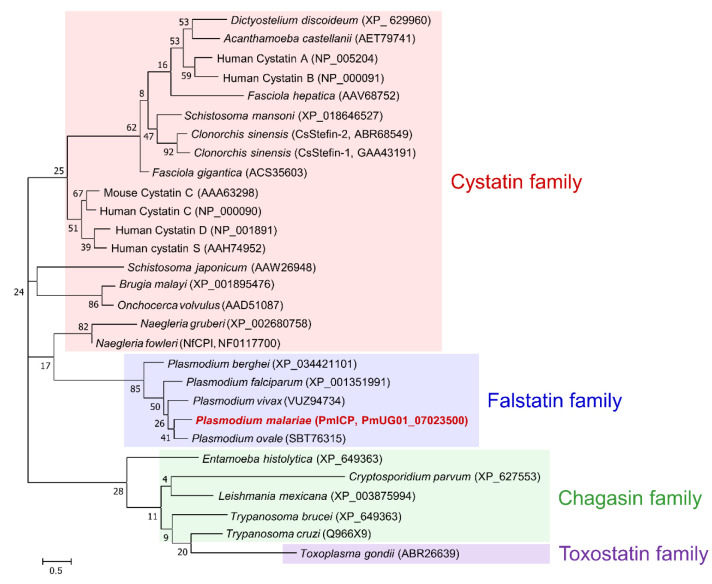
Phylogenetic analysis. The phylogenetic tree was constructed based on amino acid sequences of each protein by MEGA7 (http://www.megasoftware.net; accessed on 7 March 2022) using Maximum Likelihood Estimation (MLE) via Jones-Taylor-Thornton model with 1000 bootstrap replications. Plasmodial ICPs are clustered into a distinct clade of falstatin family separated from cystatin, chagasin, and toxostatin family proteins.

The PmICP gene was 1086 bp in length, encoding 361 amino acids with a predicted molecular mass of 40.3 kDa. Analysis of deduced amino acid sequences of PmICP revealed that PmICP had a typical N-terminal signal peptide of 20 amino acids (MNILNFLFILCSSTAFLTKC). Multiple sequence alignment analysis revealed that PmICP showed moderate sequence identities with plasmodial ICPs (31.97–46.63%), but low sequence identity with chagasin (8.56%) and cryptostatin (5.49%) (Figure 1). The predicted size of PmICP was similar to those of its orthologous ICPs from other *Plasmodium* species, but much larger than chagasin family proteins such as chagasin and cryptostatin. Four loops, L0, L2 (BC), L4 (DE), and L6 (FG), known to bind to the target enzymes [17] were well conserved in all plasmodial ICPs, although they showed sequence variations. Phylogenetic tree analysis also revealed that PmICP and plasmodial ICPs formed a different clade distinguished from cystatin, chagasin, and toxostatin family proteins (Figure 2). PmICP was the most closely related to PoICP.

### 2.2. Expression and Purification of Recombinant PmICP

**Figure 3:**
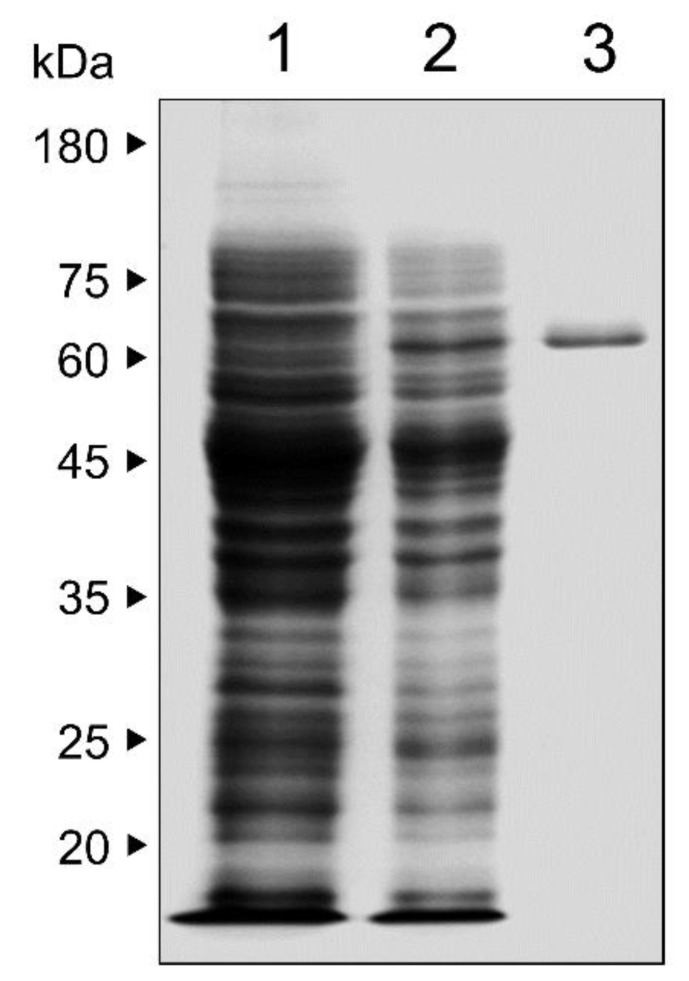
Expression and purification of recombinant PmICP. Recombinant PmICP was expressed in *Escherichia coli*, purified via glutathione agarose chromatography, and analyzed by 12% SDS–PAGE. Lane 1, non-induced *E. coli* lysate; lane 2, IPTG-induced *E. coli* lysate; lane 3, purified recombinant PmICP.

The fragment of PmICP lacking N-terminal signal peptide sequence was cloned and expressed in *E. coli*. The recombinant PmICP was expressed as a soluble protein with an approximate molecular weight of 67 kDa, which matched well with the predicted molecular weight of PmICP (38.8 kDa) and glutathione S-transferase (GST) tag (28.4 kDa) (Figure 3).

### 2.3. Biochemical Properties of PmICP

**Figure 4:**
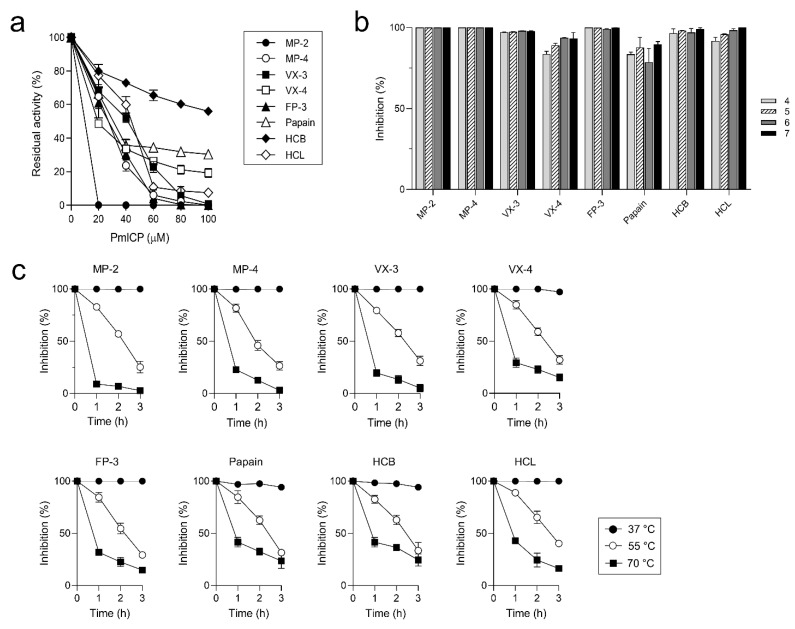
Inhibitory activity of PmICP. (**a**) Inhibition profiles of PmICP against diverse cysteine proteases. (**b**) Effect of pH. PmICP was incubated with each enzyme in different pH buffers for 20 min, after which the residual enzyme activity was assayed. (**c**) Thermal stability. PmICP was pre-incubated in 50 mM sodium acetate (pH 6.0) at 37 °C, 55 °C or 70 °C for the indicated time periods. The residual inhibitory activity of PmICP for each enzyme was assayed. All experiments were performed in triplicate and the mean and SD values were calculated.

Inhibitory activities of PmICP against several cysteine proteases including falcipain family enzymes (MP-2, MP-4, VX-3, VX-4, and FP-3), papain, HCB, and HCL were determined. PmICP inhibited these enzymes in a dose-dependent manner. However, its overall inhibitory activity was much stronger against falcipain family enzymes except VX-4 than against papain and human cathepsins (Figure 4a). These results indicated that PmICP was able to inhibit diverse cysteine proteases from different species of organisms, but showed stronger inhibitory activities against plasmodial enzymes. Inhibition assay at different pH conditions revealed that the inhibitory activity of PmICP was not greatly affected by pH as its inhibitory activities against these enzymes were similar at a broad range of pH values (Figure 4b). PmICP was stable at 37 °C, however, it rapidly lost its inhibitory activity at 55 °C and 70 °C (Figure 4c). This suggest that PmICP is a thermo-labile protein.

### 2.4. PmICP as a Regulatory Molecule of MPs 

**Figure 5:**
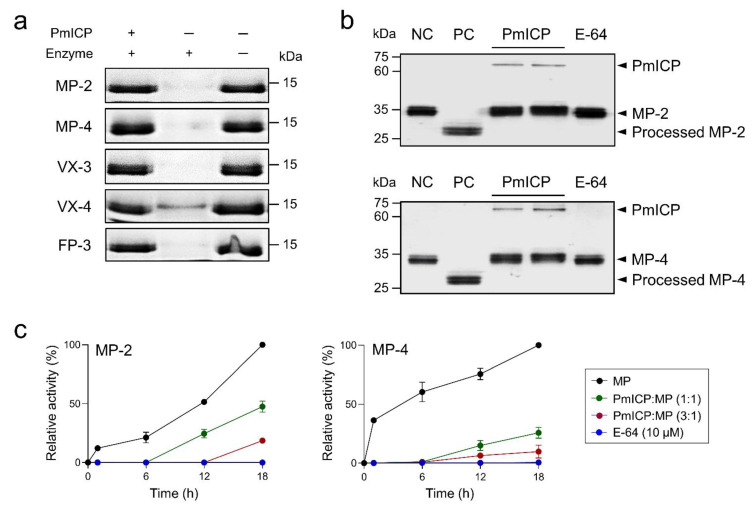
Inhibition of hemoglobin hydrolysis and maturation of MPs. (**a**) Inhibition of hemoglobin hydrolysis by MPs, VXs, and FP-3. Each fully activated enzyme (50 nM) was incubated either with or without PmICP (50 nM) at room temperature for 30 min. Human hemoglobin (1 mg/mL) was added to the mixture and incubated at 37 °C for 3 h. Samples were analyzed by 15% SDS–PAGE. (**b**) Regulation of maturation of MPs. Each MP (10 μg) was pre-incubated with PmICP (10 μg) or E-64 (10 μM), activated, and analyzed by 12% SDS–PAGE. NC, negative control before processing; PC, positive control after processing without pre-incubation neither PmICP nor E-64. (**c**) Regulation of maturation process of MPs. Each MP was processed to mature enzyme in the presence or absence of PmICP (1:1 or 3:1 ratio for MP) or E-64 (10 μM). Aliquots were taken at indicated time points and assayed for enzyme activity. Enzyme activity was assayed with Z-RR-MCA and Z-LR-MCA for MP-2 and MP-4, respectively.

PmICP effectively inhibited hemoglobin hydrolysis activities of falcipain family enzymes including MP-2 and MP-4 (Figure 5a). The regulatory effect of PmICP on the processing of MPs into mature forms was also analyzed. Each MP was processed into mature enzyme via autocatalytic processing, resulting in a time-dependent increase of enzyme activity. SDS-PAGE analysis indicated the autocatalytic processing of MPs into mature enzymes was significantly inhibited by PmICP (Figure 5b). The processing of both enzymes was also effectively down-regulated by PmICP (Figure 5c).

## 3. Discussion

PmICP shared similar structural features with its orthologous proteins from other *Plasmodium* species. These proteins have different molecular features compared to other classes of cysteine protease inhibitors in that they show the absence or presence of motifs that are well conserved in cysteine protease inhibitors. Consistent with other plasmodial ICPs in the falstatin family, PmICP also had a C-terminal chagasin-like domain with a longer N-terminal region, resulting in a large protein size of approximately 40 kDa. Four evolutionary conserved loops in plasmodial ICPs, L0, L2 (BC), L4 (DE), and L6 (FG), were also tightly conserved in PmICP, albeit minor amino acid differences in these loops were identified compared to other plasmodial ICPs. Three domains (L2, L4, and L6) found in chagasin-family proteins are known to bind directly to the active site of target enzymes [17,18]. They were commonly found in plasmodial ICPs. However, an additional fourth loop, L0, which is absent in chagasin family proteins, but is typically conserved in all plasmodial ICPs [17], is present in PmICP.

Recombinant PmICP showed a broad range of inhibitory activities against falcipain-2/3 family plasmodial enzymes, papain, HCB, and HCL. However, the overall inhibitory activity of PmICP was greater against plasmodial enzymes than against others. Broad range inhibitory activities of PmICP for falcipain family enzymes from different *Plasmodium* species suggest structural conservations of plasmodial ICPs and potent target enzymes in *Plasmodium* parasites. The underlying molecular mechanism for the preferred inhibitory activity of PmICP for falcipain family enzymes is currently unclear. Further investigation is necessary. Interestingly, unlike falstatin that does not inhibit cathepsin B probably due to inability to displace the occluding loop of the enzyme [14], PmICP showed a partial inhibitory activity against HCB, suggesting its plausible regulation functions for host cysteine proteases. VX-4 was not effectively inhibited by PmICP compared to other falcipain family enzymes and HCL. VX-4 has unique substrate preferences different from other falcipain family enzymes due to structural difference in S2 pocket of the enzyme [11]. It may cause incomplete or partial inhibition of the enzyme by PmICP. PmICP lost its activity rapidly at a high temperature, which was distinct from other thermo-stable cysteine proteases inhibitors such as cystatin and chagasin family proteins [19,20,21].

Biological roles of PmICP are currently unclear. However, highly similar structural and biochemical properties of PmICP with other falstatin family plasmodial ICPs suggest its functional relevance with these proteins. Up to now, approaches to determine biological functions of falstatin family ICPs in in vitro culturable or in vivo maintainable species of *Plasmodium* parasites have been tried. Falstatin facilitates the invasion of merozoites to erythrocytes by preventing deleterious activity of falcipains released from ruptured schizonts or other host enzymes that could interfere with the invasion process [14]. ICP of *P. berghei* (PbICP) is likely to play critical roles in hepatocyte invasion by sporozoites and block host hepatocyte apoptosis [15]. PbICP has also been proposed to play essential roles for sporozoite maturation and motility in mosquito vectors and for parasite development in hepatocytes and erythrocytes [22,23]. ICP of *P. yoelii* (PyICP) plays pivotal role in the regulation of yoelipain-2 during blood-stage development. It is likely to play a role in liver stage-hepatocyte interactions at the time of exoerythrocytic merozoite release [16]. To understand biological roles of PmICP, relevant studies such as analyses of expression patterns and localization of PmICP in different developmental stages of *P. malariae* and interactions of PmICP with MPs are necessary. Unfortunately, there is a great technical hurdle to perform these experiments in the *P. malariae* since an in vitro cultivation method of the parasite has not been established. Effective inhibition of MPs activity and regulation of maturation of the MPs by PmICP suggest its potential role in modulation of MPs, which might be essential for successful blood-stage development of the parasite. Potent inhibitory activities of PmICP against human enzymes HCL and HCB at broad ranges of pH also suggest its plausible functions in regulation of host cysteine proteases, which in turn may contribute to parasite’s development and enhanced survival in the host.

## 4. Materials and Methods

### 4.1. Cloning of a Gene Encoding PmICP

A gene encoding PmICP (PmUG01_07023500) was identified by data mining the *P. malariae* genomic resource (https://plasmodb.org/plasmo/app; accessed on 10 June 2020). Specific primers to amplify the gene were designed with the oligonucleotide sequences: 5′-ATAATAGAACGTTTACTTTTGATATAG-3′ and 5′-TTATGCTACATTCAAATGGAGAATTTT-3′. Polymerase chain reaction (PCR) was performed with the thermal condition: 94 °C for 4 min, 30 cycles at 94 °C for 1 min, 50 °C for 1 min, and 72 °C for 1 min, followed by a final extension at 72 °C for 10 min. Genomic DNA of *P. malariae* were prepared from clinical isolates collected in our previous study [24]. The PCR product was purified from the gel and ligated into the T&A cloning vector (Real Biotech Corporation, Banqiao City, Taiwan). The ligation mixture was transformed into *Escherichia coli* DH5α competent cells and the positive clones were selected by colony PCR. Nucleotide sequence of the cloned gene was analyzed by automatic DNA sequencing. Primary structure of the deduced amino acid sequences of PmICP was analyzed with DNASTAR package (DNASTAR, Madison, WI, USA), PSORT (http://www.psort.org/; accessed on 4 September 2020), and Signal P (http://www.cbs.dtu.dk/services/SignalP/; accessed on 4 September 2020). The phylogenetic tree was constructed based on the sequences of cysteine protease inhibitors from other species of *Plasmodium*, *Cryptosporidium parvum* (Cryptostatin: GU433606), and *Trypanosoma cruzi* (Chagasin: Q966X9) by MEGA7 (http://www.megasoftware.net; accessed on 7 March 2022) using Maximum Likelihood Estimation (MLE) via the Jones-Taylor-Thornton model with 1000 bootstrap replications.

### 4.2. Expression and Purification of Recombinant PmICP

The fragment of PmICP lacking the N-terminal signal peptide of 20 amino acid lengths was amplified with specific primers: 5′-GGATCCAATAGAACGTTTACTTT- TGATATA-3′ contained 5′ *Bam*HI site and 5′-GTCGACTTATGCTACATTCAAATGGA- GAAT-3′ harbored 5′ *Sal*I site. The PCR product was cloned into T&A cloning vector (Real Biotech Corporation) and transformed to *E. coli* DH5α. The resulting plasmid was digested with appropriate restriction enzymes, ligated into pGEX-6P-1 expression vector (Cytiva, Marlborough, MA, USA), and transformed into *E. coli* BL21 (DE3). Expression of recombinant PmICP was induced by adding 1 mM of isopropyl-1-thio-β-_D_-galactopyranoside (IPTG), and the *E. coli* was cultured at 16 °C overnight with shaking at 230 rpm for aeration. The cells were harvested by centrifugation at 7000 rpm at 4 °C for 15 min and suspended in lysis buffer (50 mM Tris (pH 8.0), 150 mM KCl, 0.1% Triton X-100, 1 mM DTT, and 1 mg/mL lysozyme). The suspended bacteria were sonicated 10 min with the pulse of 10 s on/10 s off on ice, and centrifuged at 12,000 rpm at 4 °C for 30 min. The recombinant protein was purified from the supernatant by glutathione agarose (Thermo Fisher Scientific, Waltham, MA, USA) following the manufacture’s instruction. The purification and purity of recombinant PmICP were determined by 12% sodium dodecyl sulfate–polyacrylamide gel electrophoresis (SDS–PAGE). The concentration of recombinant PmICP was determined by BCA protein assay kit (Thermo Fisher Scientific) following the manufacturer’s protocols.

### 4.3. Inhibitory Activity Assay

Inhibitory activity of recombinant PmICP against cysteine proteases was analyzed by measuring the residual enzyme activity after incubation of each enzyme with PmICP [21,25]. Cysteine proteases used in this study were papain (Sigma, St. Louis, MO, USA), human cathepsin B (HCB; Sigma), human cathepsin L (HCL; Sigma), malapain-2 (MP-2), malapain-4 (MP-4), vivapain-3 (VX-3), vivpapain-4 (VX-4), and falcipain-3 (FP-3). Recombinant proteins of MP-2, MP-4, VX-3, VX-4, and FP-3 were produced, refolded, and activated as described previously [10,11,13,26]. The concentration of each enzyme was determined via active site titration with trans-epoxy-succinyl-_L_-leucylamido(4-guanidino) butane (E-64; Sigma). Each enzyme (20 nM) was incubated with different concentrations of PmICP (0–100 nM) in 50 mM of sodium acetate (pH 5.5) at room temperature for 20 min. Substrate solution was then added to the mixture, and the residual enzyme activity was determined by measuring the release of fluorescence (excitation at 355 nm and emission at 460 nm) at room temperature for 30 min with a Fluoroskan Ascent FL (Thermo Fisher Scientific). Benzyloxycarbonyl-_L_-leucyl-_L_-arginine 4-methyl-coumaryl-7-amide (Z-LR-MCA; Peptide International, Louisville, KY, USA) was used as a substrate for papain, HCB, HCL, MP-4, VX-3, VX-4, and FP-3, while Z-_L_-arginyl-_L_-arginine MCA (Z-RR-MCA; Peptide International) was used for MP-2. Substrate solution was composed of 50 mM sodium acetate (pH 5.5), 10 mM dithiothreitol (DTT), and 10 nM of appropriate peptide substrate.

### 4.4. Characterization of Biochemical Properties of PmICP

Effect of pH on the inhibitory activity of PmICP was analyzed by incubating PmICP (50 nM) with the same concentration of each cysteine protease in different pH buffers (50 mM sodium acetate (pH 4.0–5.0), 50 mM sodium phosphate (pH 6.0), or Tris-HCl (pH 7.0)) at room temperature for 20 min followed by measuring residual activity of each enzyme using the assay method described above. Enzyme without incubating with PmICP at each pH value was used as positive control. Thermal stability of PmICP was determined by incubating PmICP at different temperatures (37 °C, 55 °C and 70 °C) in 50 mM sodium acetate (pH 6.0) for 0–3 h. The samples were cooled on ice, and the residual inhibitory activities of aliquoted samples against each enzyme were determined as described above. In all assays, E-64 was used as a control inhibitor. All the assays were performed in triplicate, and the mean and standard deviation (SD) of the values from the individual experiments were calculated.

### 4.5. Inhibition of Hemoglobin Hydrolysis by MPs

Inhibitory activity of PmICP to human hemoglobin hydrolysis by plasmodial cysteine proteases was also analyzed. Briefly, 50 nM of each plasmodial cysteine protease (MP-2, MP-4, VX-3, VX-4, or FP-3) was incubated either with or without 50 nM of PmICP in 50 mM sodium acetate (pH 5.5) at room temperature for 30 min. Human hemoglobin (1 mg/mL; Sigma) was then added to the mixture and incubated at 37 °C for 3 h in the presence of 1 mM DTT. For negative control, only hemoglobin (1 mg/mL) was applied with the same procedure. The reactions were stopped by adding 5× SDS–PAGE sample buffer followed by boiling. The samples were analyzed by 15% SDS–PAGE.

### 4.6. Regulatory Activity of PmICP on Maturation of MPs

Regulatory activity of PmICP on the maturation process of MPs was analyzed using a modified method as described previously [27]. In brief, recombinant MP-2 and MP-4 were produced and refolded as described previously [13]. These refolded MP-2 and MP-4 (each 10 μg) were further processed in the presence or absence of PmICP (10 μg or 30 μg) or E-64 (10 μM) at room temperature for 18 h, respectively. Aliquots were obtained from each mixture every 6 h and used to analyze enzyme activity. Enzyme activity was assayed with Z-RR-MCA and Z-LR-MCA for MP-2 and MP-4, respectively. Regulatory function of PmICP on maturation of MPs were also analyzed by SDS–PAGE. Refolded MP-2 and MP-4 (each 10 μg) were pre-incubated with either PmICP (10 μg) or E-64 (10 μM) at room temperature for 1 h and incubated in sodium acetate (pH 5.0) at 37 °C for 2 h, respectively. The proteins were concentrated with Centricon Plus (cut-off 10 kDa; Merck Millipore, Burlington, MA, USA) and analyzed by 12% SDS–PAGE.

## 5. Conclusions

Functional expression and characterization of PmICP can widen our knowledge on the nature of the protein. Production of recombinant PmICP offers a valuable material for further studies to investigate PmICP-MPs interaction and rational design of specific inhibitors for effective antimalarial drugs based on cysteine proteases of malaria parasites. Considering highly similar structural and biochemical features of PmICP with orthologous falstatin family proteins from other *Plasmodium* species, PmICP is likely to have functional relevance with these proteins. Further comprehensive studies such as molecular modeling to understand underlying molecular interactions between PmICP and MPs and efforts to determine the biological significance of PmICP in both parasite and host are necessary.

## Data Availability

The data supporting the conclusions of this article are provided within the article. The original data in the present study are available from the corresponding authors upon request.
